# Comparison of different approaches applied in Analytic Hierarchy Process – an example of information needs of patients with rare diseases

**DOI:** 10.1186/s12911-016-0346-8

**Published:** 2016-09-09

**Authors:** Frédéric Pauer, Katharina Schmidt, Ana Babac, Kathrin Damm, Martin Frank, J.-Matthias Graf von der Schulenburg

**Affiliations:** Center for Health Economics Research Hannover (CHERH), Leibniz University of Hannover, Otto-Brenner-Straße 1, Hannover, 30159 Germany

**Keywords:** Decision-making, Analytic Hierarchy Process, Rare disease, Patient priorities, Internet homepage

## Abstract

**Background:**

The Analytic Hierarchy Process (AHP) is increasingly used to measure patient priorities. Studies have shown that there are several different approaches to data acquisition and data aggregation. The aim of this study was to measure the information needs of patients having a rare disease and to analyze the effects of these different AHP approaches. The ranking of information needs is then used to display information categories on a web-based information portal about rare diseases according to the patient’s priorities.

**Methods:**

The information needs of patients suffering from rare diseases were identified by an Internet research study and a preliminary qualitative study. Hence, we designed a three-level hierarchy containing 13 criteria. For data acquisition, the differences in outcomes were investigated using individual versus group judgements separately. Furthermore, we analyzed the different effects when using the median and arithmetic and geometric means for data aggregation. A consistency ratio ≤0.2 was determined to represent an acceptable consistency level.

**Results:**

Forty individual and three group judgements were collected from patients suffering from a rare disease and their close relatives. The consistency ratio of 31 individual and three group judgements was acceptable and thus these judgements were included in the study. To a large extent, the local ranks for individual and group judgements were similar. Interestingly, group judgements were in a significantly smaller range than individual judgements. According to our data, the ranks of the criteria differed slightly according to the data aggregation method used.

**Conclusions:**

It is important to explain and justify the choice of an appropriate method for data acquisition because response behaviors differ according to the method. We conclude that researchers should select a suitable method based on the thematic perspective or investigated topics in the study. Because the arithmetic mean is very vulnerable to outliers, the geometric mean and the median seem to be acceptable alternatives for data aggregation. Overall, using the AHP to identify patient priorities and enhance the user-friendliness of information websites offers an important contribution to medical informatics.

**Electronic supplementary material:**

The online version of this article (doi:10.1186/s12911-016-0346-8) contains supplementary material, which is available to authorized users.

## Background

The number of studies measuring patient priorities by using the Analytic Hierarchy Process (AHP) has increased significantly in the last few years [1]. The AHP was developed by Thomas L. Saaty in the 1970s to solve complex problems of multiple criteria decision-making [2], based on the idea that it is more reliable to judge the relative importance of several criteria with the help of respective pairwise comparison in a hierarchical structure than to judge their absolute importance [3]. The method was originally applied in the marketing sector and later in healthcare research. In addition, the AHP can be used to relate subjective criteria, which can be both quantitative and qualitative. As implied, it has been demonstrated that the AHP is a useful method for healthcare delivery as well as medical informatics decision-making [1, 4–7]. In this study, we ranked the information needs of people having a rare disease and their relatives using different AHP methods. This ranking of information needs is then transferred accordingly to display information categories on a web-based information portal about rare diseases in Germany. Because the available space on a user-friendly website homepage is restricted, the most important categories should be more accessible than less important categories. To present information categories on this website according to the user’s priorities, this paper consulted both experts in medical informatics and patient-reported outcomes.

Today, approximately 4 million people in Germany suffer from rare diseases. The level in the United States is similar to that in Europe, with approximately 30 million people living with rare diseases. It is estimated that 400 million people worldwide suffer from a rare disease. Currently, international definitions of rare diseases vary greatly. For example in the EU, a disease is considered rare if it affects fewer than one in 2000 citizens, whereas in the United States a disease is considered rare if it affects fewer than 200,000 people, or about one in 1500 people [8, 9]. To improve patients’ well-being, a national action plan for people with rare diseases was adopted by the Federal Government in Germany in 2013 that is supposed to coordinate national efforts invested in rare diseases. The establishment of a rare diseases information portal is one component of a broader set of planned measures, which includes 52 policy proposals [10]. Although conditions may differ significantly, patients having rare diseases and their relatives frequently face similar challenges [10, 11], which include protracted diagnosis processes as well as a deficient information base. To address these deficiencies, both medical experts and experts on medical informatics consider it relevant to assess the priorities of the (potential) patients and relatives.

As part of the development of an information portal for rare diseases, we used the AHP to identify the importance of several information types, e.g., information about therapy and social-legal advice. However, there are no best practices or a common gold standard available for applying the methods [1]. More precisely, it is noticeable that there are several methodological differences in the published studies concerning data acquisition and aggregation [1]. In some studies, single participants were interviewed (e.g. [12–14]), whereas in others, group discussions were used to analyze the priorities (e.g. [15, 16]). It therefore remains unknown which data acquisition method is more suitable for the AHP. To determine whether two methods (individual and group decisions) yield the same outcomes, we implemented them separately. The goals of this study were on the one hand to analyze the different influences of individual and group judgements on data acquisition, and on the other hand, to examine the different effects on the AHP results of using the arithmetic and geometric mean as well as the median for the data aggregation. We also discuss the degree to which the results of this study can be transferred to other disciplines. Finally, we fulfill our objective of providing a recommendation on choosing appropriate methods for further studies using the AHP.

## Methods

### Participants

Patients suffering from a rare disease were eligible to participate in the study. In addition, the relatives of these patients, for example, the parents of a child suffering from such a disease, were eligible to participate. The inclusion of both patient and relatives is necessary because many patients suffering from a rare disease are diagnosed as children, and the information priorities of the parents appear as a proxy for the children’s priorities. Moreover, both patients and relatives will use the information portal. Patients were excluded if they were unable to concentrate continuously on the questionnaire or did not adequately understand the German language. Participants were recruited by the Freiburg Centre for Rare Diseases (Medical Center of the University Freiburg, Germany) and through rare disease self-help groups.

### Analytic Hierarchy

The AHP is a stepwise problem-solving procedure. First, the decision-makers have to construct a hierarchical structure of the criteria. To achieve this, the multiple criteria decision problem must be broken down into its component parts [17]. The information needs of people suffering from a rare disease were identified by an Internet research study, including a review of already existing websites providing information on rare diseases. Furthermore, a preliminary qualitative study, the subjects of which were patients suffering from a rare disease, yielded important findings about the wording of the identified items that were regarded as the defined targets. We designed a three-level hierarchy by grouping these items into information fields and information types.

**Fig. 1:**
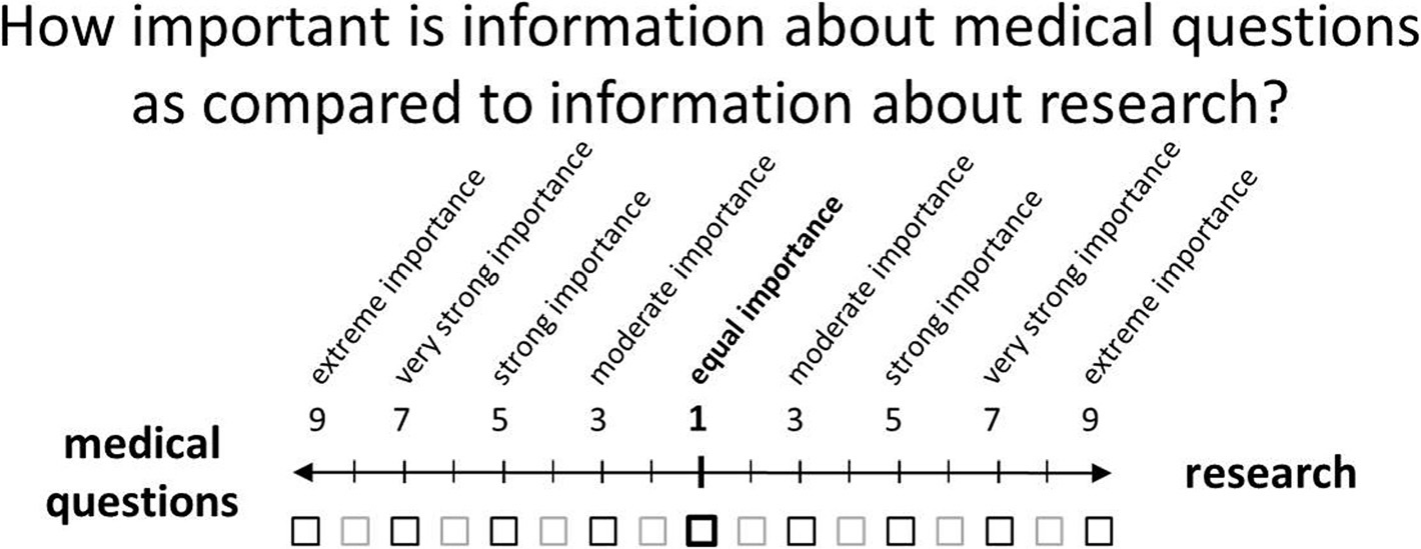
Example of a pairwise comparison on a 9-point-scale

The next step was to analyze the priorities. Patients and relatives were asked to compare every two information fields in the second level at each time with respect to the target. The information types in the third level were also compared pairwise with respect to the corresponding information field. Participants were asked to judge the importance of one endpoint as compared with another on a 9-point scale [18]. The participants also received printed ranking cards with the information fields and information types, which helped them provide consistent answers to the pairwise comparison questions. One example of a pairwise comparison is displayed in Fig. 1. It can be seen that “1” indicates that the two endpoints are of equal importance and “9” that the importance of one endpoint is extremely different from that of the other. Based on matrices of the pairwise comparisons, the standard AHP eigenvector method was used to calculate the patient’s priorities using Microsoft Windows Excel [18]. The questionnaire used in the studies is avaliable as Additional file 1.

The final operation was consistency verification, which is listed as one of the key benefits of the AHP [19]. Saaty demonstrated that the consistency ratio (CR) can be calculated using the consistency index and the random index [18]. The CR value of a perfectly cardinal consistency matrix is 0. The CR value reflects the internal consistency of an observed set of judgements, and CR ≤ 0.2 has been determined to be an acceptable level of consistency [20, 21]. The results of participants who answered consistently were included in the analyses. Finally, the priorities of individual participants were aggregated to analyze the priorities of all the participants. The different data acquisition and aggregation methods are described in the following section.

### Data acquisition

**Fig. 2:**
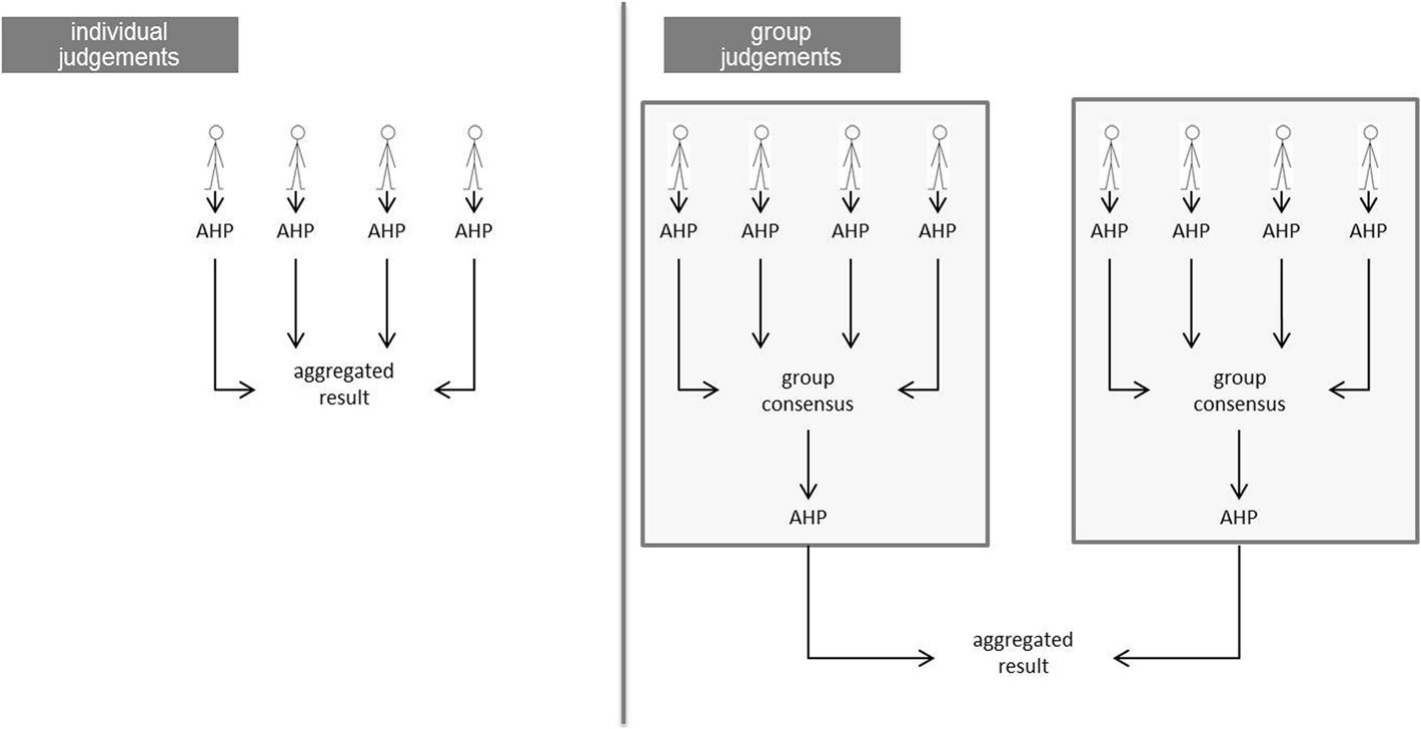
Individual and group Analytic Hierarchy Process

For data acquisition on individual decision-making, patients and relatives were interviewed. The interviews were conducted by telephone or in a face-to-face situation in a place familiar to the participant. In the case of telephone interviews, the AHP questionnaire was mailed to the participants a few days before the appointment. At the beginning of the interview, the structure of the AHP and the broad outline of the method, as well as all the quality criteria, were explained. Thereafter, the participants completed a guided AHP. Finally, the calculated individual weights (priorities of each criterion) were aggregated (Fig. 2) when the answers were consistent, as described above.

The same AHP questionnaire was used for the face-to-face group discussions. The group meetings were held at the Universities of Hannover, Frankfurt am Main, and Freiburg im Breisgau. After the interviewer presented a description of the structure and method of the AHP, each group member judged the relative priorities of each comparison. Then, the individual judgements (on a 9-point scale) were gathered and displayed anonymously on a screen. The group members discussed each pairwise comparison, as well as the rationales behind the individual judgements. Finally, for each pairwise comparison, a common group decision (consensus) was reached. The calculated group priorities were aggregated with all the other group priorities (Fig. 2) when the answers were consistent, as described above. The distribution of the priorities of individual and group weights was analyzed in separate box plots for each category using the statistics software R.

### Data aggregation

Priorities can be aggregated using the arithmetic mean. According to a frequently used method for aggregating the priorities of individuals into a consensus rating, we also used the geometric mean [21–23]. In addition, we used the median to calculate the mean value of the priorities. The median divides the data set into two equal parts and indicates the mean value. The individual priorities were aggregated using each of these methods independently to consider the different distributions resulting from the different methods. These results are presented in the “Data aggregation” subsection of the Results section.

## Results

### Participants

Thirty-six patients suffering a rare disease and four relatives (*n* = 40) having an average age of 50.7 years (ages ranged from 18 to 74 years) participated in the AHP in which the individual method was applied. In addition, for the group method, eight patients and three relatives were divided into three groups having a size of three or four participants. The average age of the group members was 52.2 years (ages ranged from 40 to 85 years). There were more female than male members in both populations. The average ages are relative high for both samples because adult relatives acted as a proxy for their children. Related to the issue, these relatives would search for information about rare diseases in the information portal. The following numbers of patients were suffering from the following rare diseases (note: the assignment to the orpha.net classification of rare diseases is not clearly regulated): rare skin diseases (five patients/two relatives), rare tumors (six patients), rare metabolic diseases (four patients), rare immunodeficiencies (seven patients), rare eye diseases (one patient), rare lung diseases (two patients/one relative), rare muscular diseases (two patients), rare blood count disorders (seven patients), rare genetic diseases (four patients/one relative), rare kidney diseases (two patients), rare skeletal dysplasia (one relative) and rare neurological diseases (four patients/two relatives). The demographic statistics of all the participants are displayed in Table 1. In addition to the information in the table, the average age at the time of diagnosis was 33.8 years for the individual AHP and 34.3 years for the group AHP; some patients were diagnosed at birth. The patients in the individual AHP had lived an average of 16.9 years since the diagnosis of a rare disease, and the group members had lived an average of 19 years since diagnosis. The marital status of the study population of the individual AHP was as follows: 27 of the 40 participants declared that they were married, six were divorced, and seven were living without a partner. Five of the group members were living with a partner, two were widowed, and four had no partner.

**Table 1:** Demographic statistics of the study population

Variable	Characteristics	Individual		Group	
		Frequency	Rate	Frequency	Rate
Sex	male	11	27.5 %	4	36.4 %
	female	29	72.5 %	7	63.6 %
Age	x < 30	2	5.0 %	0	0.0 %
	30 ≤ x < 50	18	45.0 %	6	54.6 %
	50 ≤ x <70	16	40.0 %	4	36.4 %
	x > 70	3	7.5 %	1	9.1 %
Labor status	employed	17	42.5 %	6	54.6 %
	retired	11	27.5 %	2	18.2 %
	disabled	10	25.0 %	2	18.2 %
	student	1	2.5 %	0	0.0 %
	n/a	1	2.5 %	0	0.0 %
Estimated severity of the disorder	low	6	15.0 %	2	18.2 %
	medium	19	47.5 %	4	36.4 %
	high	15	37.5 %	5	45.5 %
Status	patient	36	90.0 %	8	72.7 %
	relative	4	10.0 %	3	27.3 %

### Analytic Hierarchy

**Fig. 3:**
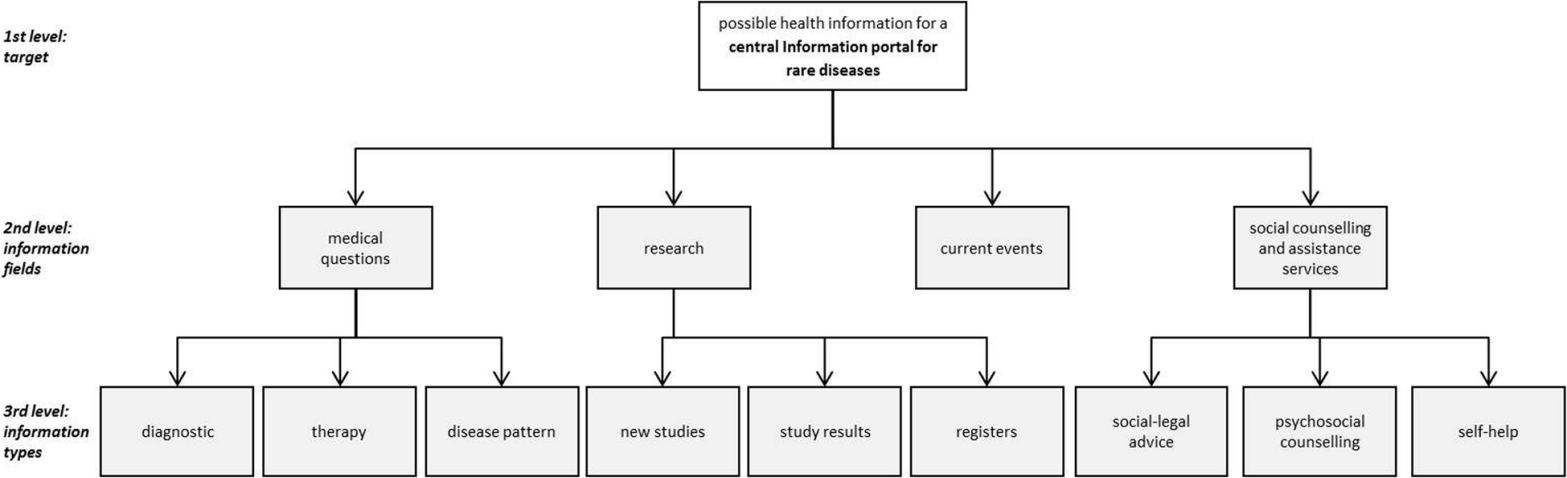
Hierarchical structure

The informational content of 300 websites maintained by providers of information about rare diseases was analyzed to identify the important items. These items were structured into a three-level hierarchy by grouping them into information fields and information types. We included four information fields: *medical questions, research*, *current events*, and *social counselling and assistance services*. Subsequently, we included nine information types: *diagnostics*, *therapy*, *disease pattern*, *new studies, study results*, *registers*, *social-legal advice*, *psychosocial counselling*, and *self-help*. The hierarchical structure (Fig. 3) contains the target on the first level, the information fields on the second level, and the information types on the third level. Consequently, for analyzing the priorities, 15 pairwise comparisons in each questionnaire were conducted: six comparisons of the four information fields on the second level and three times three comparisons of information types on the third level. An explanation of each information criterion was given to all participants, as shown in the Appendix.

### Consistency ratio

**Fig. 4:**
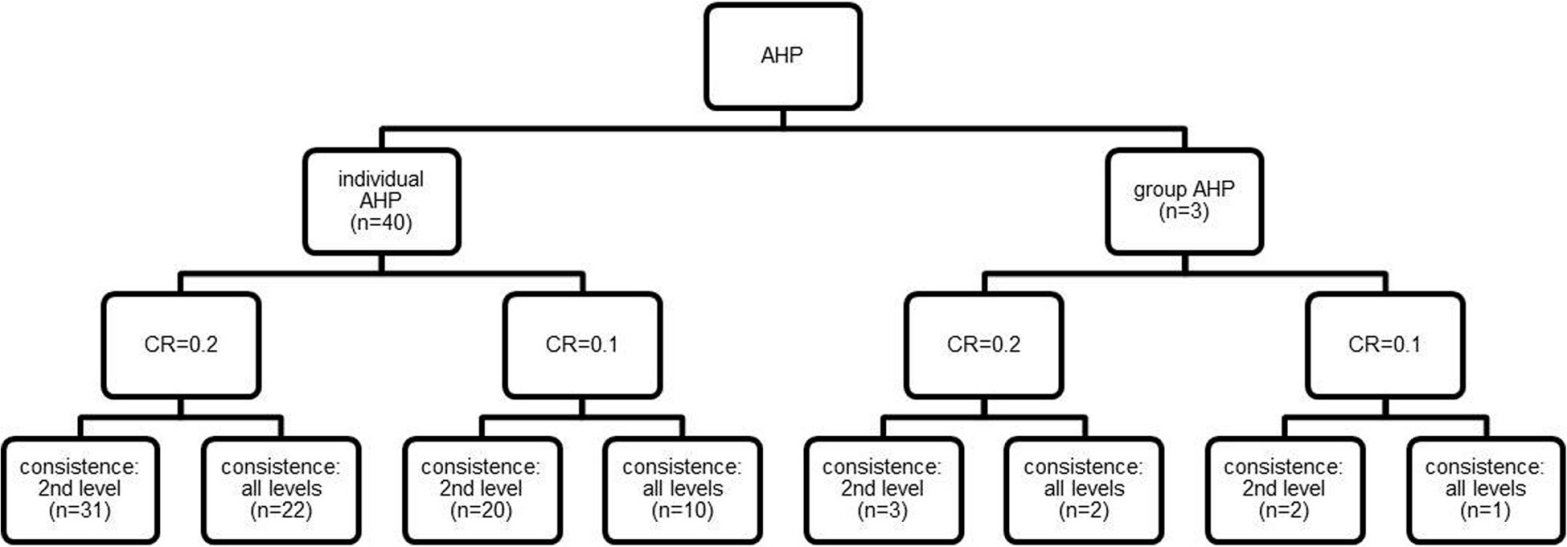
Sample sizes by different levels of consistency ratio

The study sample showed a wide range of CRs. When the acceptable CR was set at a lower level, fewer participants could be included in the analyses. Moreover, the number of included participants decreased if consistency was required at all the investigated levels. Figure 4 shows an overview of the sample sizes according to the different levels of consistency. We determined an acceptable level of consistency to be a CR of 0.2 on the second level of the hierarchy. These parameters led to 31 individual judgements and all three group judgements being included in the analysis. However, the following results differed only slightly by determining a CR of 0.1.

### Data acquisition

**Fig. 5:**
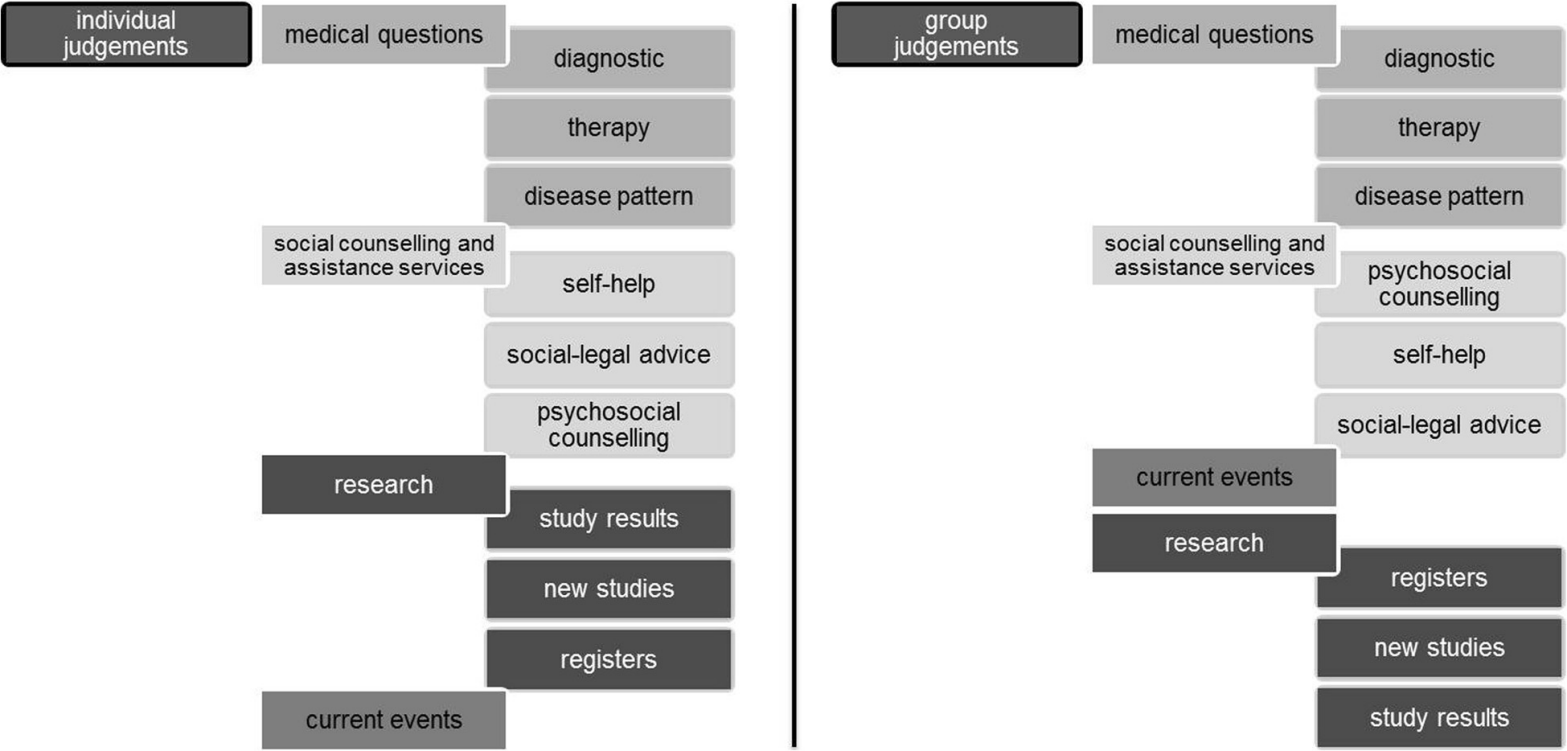
Local ranks of individual and group judgements

Further analyses were conducted by comparing individual and group priorities on the same level of consistency. The comparisons were conducted between individual and group priorities that were included in the CR = 0.2 category on the second level of the hierarchy. Figure 5 presents the corresponding local ranks of the information types (second level) and information fields (third level). To a large extent, the local ranks for individual and group judgements were similar. In both, *Information about medical questions* was the most relevant information type. In addition, the order of information fields (*diagnostic*s, *therapy,* and *disease pattern*) in this information type was the same. Furthermore, in the second rank, information about *social counselling and assistance services* can be evaluated for individual and group priorities. Moreover, we found differences between individual and group judgements: *information about current events* was ranked higher by the group participants, and the order of the information fields *registers*, *new studies*, and *study results* differed.

**Fig. 6:**
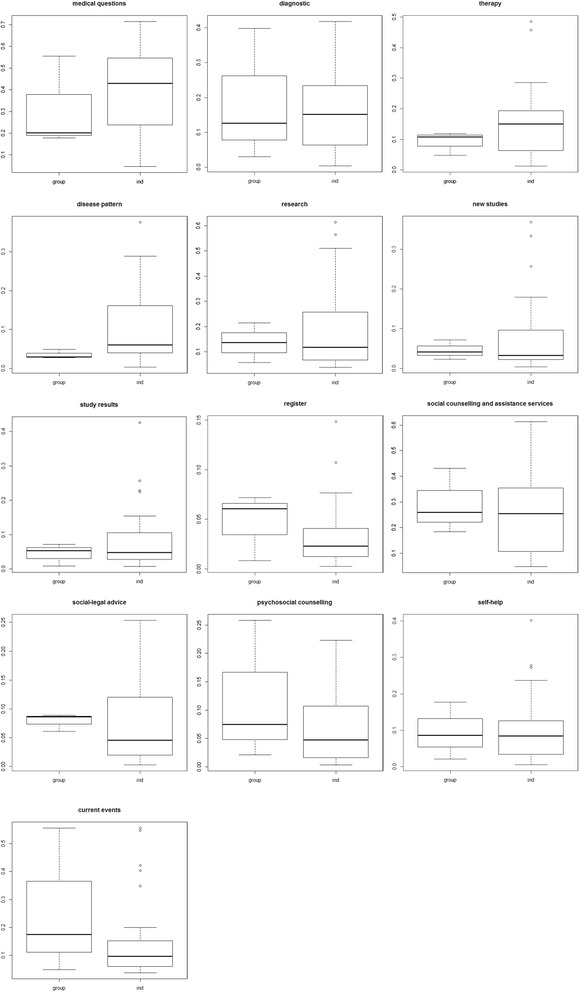
Distribution of priorities of individual and group judgements

In addition to the comparison above, we analyzed the weights of each category for the individual and group priorities separately. The (global) weights quantify the priorities and allow all the information categories to be compared. The distribution of priorities for each category is displayed in Fig. 6. For each category, the distribution of group priorities (*group*) and individual priorities (*ind*) is shown. Based on the median, the differences between the individual and group priorities were small. For example, the weight of the category *information about medical questions* was noticeably higher for individual priorities. For the category *information about registers*, the weight was higher for group priorities. Moreover, we determined that the data span from minimum to maximum was most frequently greater for the individual priorities than for the group priorities.

**Fig. 7:**
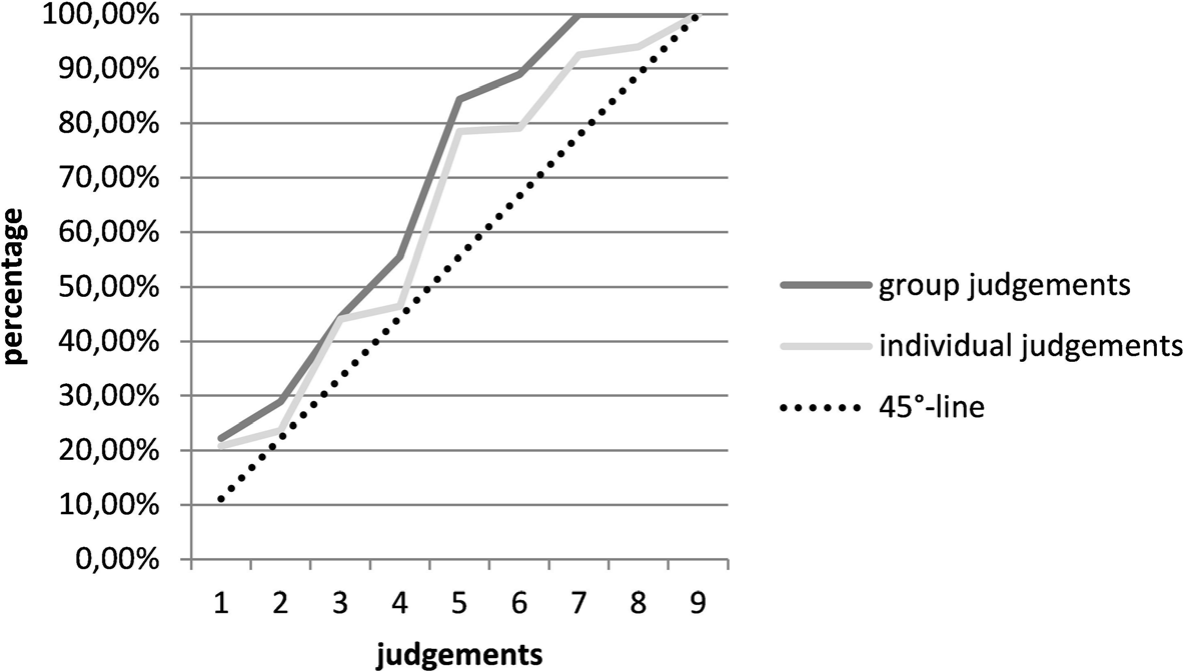
Distribution of the given answers

Furthermore, we analyzed the answers given as individual judgements compared to those given as group judgements. The cumulative relative value distribution indicates the response behavior of individuals and groups. Figure 7 shows that group judgements frequently were in a narrower range than individual judgements; in particular, most of the judgements were located between 1 = equally important and 5 = very important. Stronger priorities (7 = very strongly important to 9 = extremely important) were not used in group judgements. The 45°-line symbolizes an equal distribution of the judgements between 1 = equally important and 9 = extremely important. Statistically significant differences between individual and group judgements (*p* = 0.0027) were found using a t-test analysis.

### Data aggregation

Aggregating single priorities is required to generate a summary of the study results. Depending on the data aggregation method, the ranks of the information criteria and the corresponding weights differ slightly. An advantage of using different methods separately is that the different distributions of the data sets can be considered and results can be compared between the methods.

**Fig. 8:**
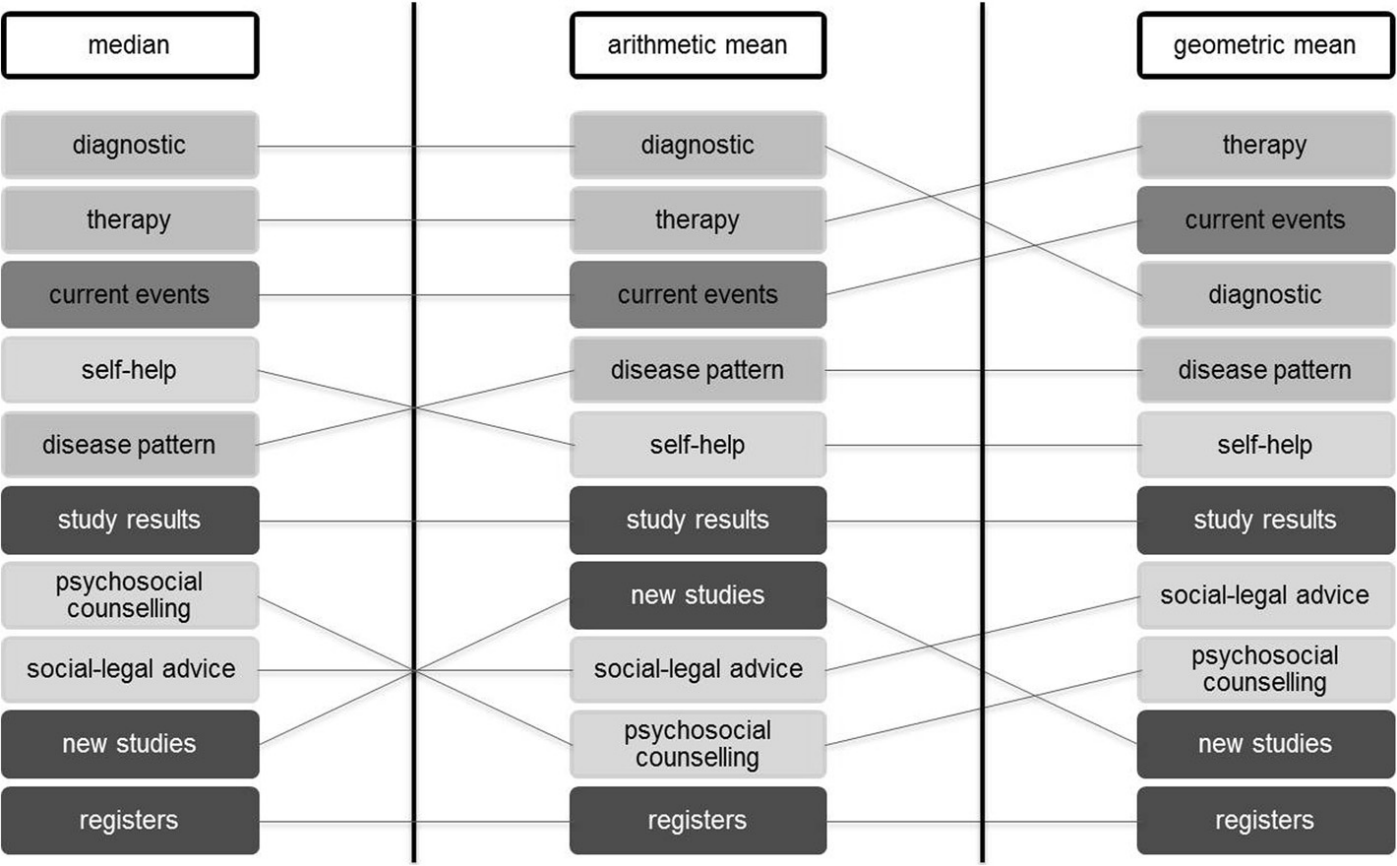
Comparison of data aggregation by median and arithmetic and geometric mean

Figure 8 shows the global ranks of the items grouped by the methods used for data aggregation (arithmetic and geometric mean, as well as the median). A comparison of the global ranks of the aggregation by the arithmetic mean with the aggregation by the geometric mean reveals that the criterion *information about diagnostic*s had a lower priority if the data were aggregated by geometric mean. The same result was obtained for *information about new studies*. Other information criteria showed the same global ranking for both aggregation methods. A comparison of the global ranks of the aggregation by median with the aggregation by arithmetic mean showed that the criteria *information about self-help* and *information about disease patterns* changed ranks, as did the criteria *information about psychosocial counselling* and *information about new studies*. In summary, according to our data, there is no strong difference between the ranking of information criteria when the data are aggregated by the median or by the arithmetic or geometric mean.

## Discussion

We have demonstrated that the AHP can be used to identify patient priorities with regard to the information needs of people having rare diseases. For this purpose, group decisions were as suitable as individual decisions. Although the local rank of the information types resulted in a similar order of individual and group decisions, their global weights varied slightly. Interestingly, we found another important aspect: group judgements were in a significantly smaller range than individual judgements. This result may be correlated with the fact that group judgements are more frequently consistent. Hence, it could conceivably be hypothesized that using smaller ranges, e.g., a 7- or 5-point scale, would lead to more consistent answers. Unfortunately, we cannot compare the response behavior with that reported in other published studies, because such an analysis was not conducted in these studies [1]. Furthermore, it can be argued that group decisions frequently represent the compromise solution of the group participants, and therefore, the group judgements are a mean of the individual judgements and consequently the group’s priorities have a more limited range. We attempted to avoid a situation in which the group participants gave only the mean of their individual judgements as their answer. Frequently, the group participants discussed the rationales behind the individual judgements and decided on a common group priority that was not the mean of the individual judgements. Sometimes, the group judgement was even outside the range of the individual minimum and maximum judgements. There are, however, other possible explanations that should be investigated in further studies.

The findings of this study suggest that there is no “gold standard” method for data acquisition. According to our data, both the individual and group methods lead to very similar results. Moreover, there is no right or wrong ranking of the priorities of information needs. Researchers should select the most suitable method using other criteria, such as the thematic perspective of the study or the properties of the goods or topics that are addressed. It can be argued that, on the one hand, for free or non-rival goods, methods that involve individual decision-making are more suitable, because there is no need for the participants to be prepared to compromise; other people will not face disadvantages or advantages because of one individual’s decision. On the other hand, group decisions are suitable for scarce or rival goods. Another aspect that should be considered is the peer pressure exerted in group discussions. The group situation can lead to particular disadvantages when intimate insights should be given in the interview, in which case, individual participants do not dare to answer truthfully or do not state their personal opinions. With regard to the implementation of the rare disease information portal or other websites, the order of information categories should not be influenced by other users. Therefore, an individual user’s priorities shall be used to identify which information categories are more important and should be more accessible on the website than less important categories. In summary, the use of patient priorities to expand the user-friendliness of information websites using the AHP offers an important contribution for medical informatics.

According to our data, aggregations by median, arithmetic mean, and geometric mean lead to very similar rankings of information criteria. Because the arithmetic mean is very vulnerable to outliers, the median and the geometric mean appear to be acceptable alternatives for data aggregation, although the differences between the two methods depend on additional factors, such as the number of criteria in the hierarchy and the number of participants. Nevertheless, comparing the analyses using different methods offers the advantage of enabling consideration of the different distributions of the data sets.

The AHP method can lead to judgements that do not meet the defined CR requirement. We determined that the use of ranking cards prior to pairwise comparison of each category may help participants answer more consistently. Furthermore, we noticed that a comparison of four aspects of a category (such as the comparison of four information fields) is more challenging for participants than a comparison of three aspects of a category (such as the comparison of three information types) in terms of cardinal consistency. This fact was used to confirm the conditions for participation in this study: patients who were unable to concentrate on the questionnaire continuously were excluded, as well as children. This participation bias may lead to a non-representative ranking of the information needs of people suffering from a rare disease. Further applications of the AHP should consider restricting the number of pairwise comparisons in each category. Moreover, by setting a CR at ≤ 0.2, we could include a sufficient number of judgements in our analysis. If we had set a lower CR value, the number of included judgements would have been lower, and consequently, the informative value of this study would have been more limited.

### Assumptions and limitations

The number of patients living with any one rare disease is limited. For this reason, we pooled patients with heterogeneous rare diseases, who frequently face similar challenges and have similar information needs. However, because of the relatively low number of participants interviewed in this study, the results may not be representative. Furthermore, a bias exists regarding the information criteria *current events*, because no information types were grouped in this information field. In addition, we attempted to minimize the interviewer bias, as well as the bias between telephone and face-to-face interviews.

## Conclusions

To the best of our knowledge, this is the first study to investigate the differences in individual and group judgements when conducting an AHP. Our study demonstrated the need for better strategies for choosing an appropriate method. Both methods led to similar outcomes; however, the response behavior differed. In brief, we demonstrated that the AHP can be used to identify the importance of several information types to people having a rare disease, and to order these information types on a website that presents information on rare diseases. Using the results of the AHP, we could rank the information needs of people suffering from a rare disease and their relatives according to their priorities. These priorities can be used to constitute information categories that are more important and should be more accessible on the website than less important categories. Overall, the use of an AHP to identify patient priorities and expand the user-friendliness of information websites offers an important contribution to medical informatics. According to our data, the use of different methods for data aggregation had no distinct influence on the ranking of the information criteria.

The strength of our study is in the transparent comparison of the different approaches applied in the AHP. The study indicates appropriate methods for conducting an AHP in other healthcare settings and in the field of medical informatics. Even if the results of the data acquisition methods do not differ, as was shown in our data, it is important that the researcher explain and justify the choice of method. We suggest that researchers select a suitable method based on the thematic perspective of the study or the properties of the goods or topics they are addressing. For example, it can be argued that group judgements should be used for studies addressing goods with limited availability. This investigation yielded important findings for subsequent studies that use the AHP method as a tool for medical decision-making and identifying patients’ priorities.

## Abbreviations

AHP, analytic hierarchy process; CHERH, center for health economics research hannover; CR, consistency ratio; Ind, individual.

## Appendix

### Definitions of the information criteria

*Medical questions:* Information that contains medical background information about rare diseases, e.g., information about diagnostics, therapy, or disease pattern.

*Diagnostics:* Information about diagnostic procedures using which a healthcare professional can identify rare diseases and make a diagnosis. In addition, contact information about specialized healthcare professionals or centers for rare diseases.

*Therapy:* Information about treatment procedures. In addition, contact information about healthcare professionals who can treat people suffering from a rare disease.

*Disease pattern:* Information about reasons for, symptoms, and progression of rare diseases.

*Research:* Information and results of scientists or pharmaceutical companies about new findings related to rare diseases.

*New studies:* Investigations of medical treatments of rare diseases that are scheduled or starting immediately for which participants are still being sought.

*Study results:* Results of current medical research.

*Registers:* Collections of disease data in the long term to improve the treatment opportunities and to monitor the distribution of the diseases.

*Current events:* Information and important appointments for public meetings where patients and affected persons can talk to healthcare staff.

*Social counselling and assistance services:* Contact data for and information about counselling centers that can help people suffering from a rare disease.

*Social-legal advice:* Here, answers can be found to questions concerned with the services of statutory health insurance, labor laws, or statutory pension funds.

*Psychosocial counselling:* Information and contact data that can provide psychosocial counselling in the case of illness-related problems of family, friends, or coworkers.

*Self-help:* Contact information about support groups of patients and close relatives.

## Additional file

Additional file 1: Questionnaire. (PDF 556 kb)
