# Supplementary material for: Comparison of different approaches applied in Analytic Hierarchy Process – an example of information needs of patients with rare diseases
Source: BMC Med Inform Decis Mak. 2016 Sep 9;16(1):117. doi: 10.1186/s12911-016-0346-8 (PMC5016921; doi:10.1186/s12911-016-0346-8)
Supplement: Additional file 1: — Questionnaire. (PDF 556 kb) [file 12911_2016_346_MOESM1_ESM.pdf]

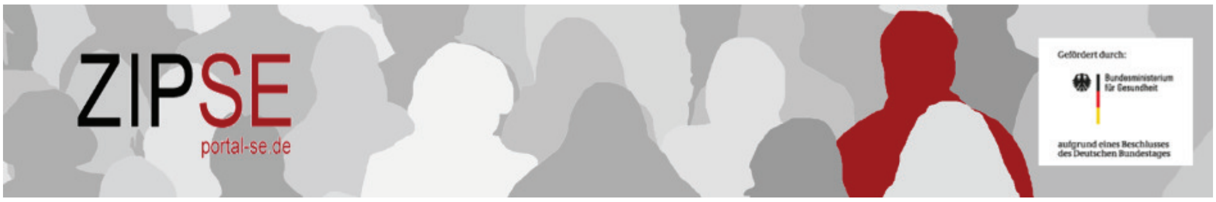

Zentrales Informationsportal für Seltene Erkrankungen:

Informationsnachfrage

Vorabinformation

Hintergrund

Ziel des Forschungsprojektes ist der Aufbau eines zentralen Informationsportals über Seltene Erkrankungen (ZIPSE), welches sowohl Betroffenen und ihren Angehörigen als auch Ärzten, Therapeuten und Pflegekräften qualitätsgesicherte Informationen bereitstellt. Dabei sollen bereits verfügbare Informationen über seltene Erkrankungen auf der zentralen Portalseite gebündelt werden. Weiterführende Informationen zum Projekt finden Sie auf der Website der ZIPSE unter: www.portal-se.de.

Ziel der Befragung

Damit Sie in Zukunft genau die Informationen auf der ZIPSE-Website finden, die Sie besonders interessieren, möchten wir Sie bitten, an der folgenden Befragung teilzunehmen und uns Ihre **persönliche Meinung** mitzuteilen. Wir möchten von Ihnen erfahren, wie wichtig für Sie persönlich verschiedene Informationen sind. Bitte fragen Sie sich hierbei, welche Informationen zum **aktuellen Zeitpunkt in Ihrer aktuellen Situation für Sie** wichtiger sind. Dabei gibt es keine falschen Antworten! Zur Vorbereitung auf diese Befragung geben wir Ihnen auf den nächsten Seiten Erklärungen zu möglichen Bereichen, aus denen Sie Information zu seltenen Erkrankungen bekommen könnten. Bitte lesen Sie sich diese sorgfältig durch.

Ihre Daten werden selbstverständlich vertraulich behandelt und ausschließlich im Rahmen des ZIPSE Projektes verwendet. Um dies zu gewährleisten, werden Ihre Angaben pseudonymisiert abgespeichert. Die Zugangsberechtigung zu den erhobenen Daten liegt ausschließlich bei den Mitarbeitern des Projektes. Es werden keine Daten an Dritte weitergegeben.

Es entsteht Ihnen kein Nachteil, falls Sie nicht an der Befragung teilnehmen möchten.
Gerne wollen wir uns schon im Vorfeld für Ihre Mühe bedanken!

Erläuterung der Informationsbereiche – Im Detail

Bitte lesen Sie nun sorgfältig die Beschreibungen der einzelnen Bereiche und Arten von möglichen Informationen für die ZIPSE-Website durch.

1. Medizinische Fragen

Unter diesem Bereich verstehen wir Informationen, die etwas mit dem medizinischen Hintergrund einer seltenen Erkrankung zu tun haben. Darunter fallen zum Beispiel Fragen zur Diagnose, der Therapie oder der Entstehung von einer seltenen Erkrankung.

a) Diagnosefindung:

- *Leistungserbringer:* Namen, Adressen, Telefonnummern, Homepages von Ärzten, die auf die Diagnose von seltenen Erkrankungen spezialisiert sind.
- *Diagnoseverfahren:* Methoden in der Medizin und ihr Ablauf, mit denen seltene Erkrankungen erkannt werden können.

b) Therapie:

- *Behandelnde Leistungserbringer:* Namen, Adressen, Telefonnummern, Homepages von Ärzten und Therapeuten, die auf die Behandlung (Therapie) von seltenen Erkrankungen spezialisiert sind.
- *Behandlungsverfahren:* Informationen, die Behandlungsmethoden von seltenen Krankheiten beschreiben (z.B. Operationen oder Medikamente).

c) Allgemeines zum Krankheitsbild:

- *Ursachen:* Hier werden die Auslöser von seltenen Erkrankungen erklärt, soweit diese bekannt sind. Zum Beispiel können genetische Defekte die Ursache sein.
- *Häufigkeiten:* Unter diesem Punkt ist zu finden, wie viele Personen von der seltenen Erkrankung betroffen sind.
- *Symptome:* An dieser Stelle werden Informationen gegeben, die Symptome oder typische Erscheinungen der Krankheit beschreiben.
- *Verlauf:* Das bedeutet, welche Veränderungen sich bei den Patienten mit der Zeit ergeben und in welcher Phase der Krankheit Veränderungen stattfinden.

2. Forschung

Der zweite große Bereich stellt Informationen zur Forschung bereit. Das bedeutet, dass es hier unter anderem Informationen dazu gibt, welche Bemühungen Arzneimittelhersteller gerade tätigen oder was Wissenschaftler Neues über seltene Erkrankungen herausfinden konnten.

a) Anlaufende Studien:
Untersuchungen von seltenen Erkrankungen, die in Planung sind bzw. gerade anlaufen und noch nach Studienteilnehmern suchen.

b) Studienergebnisse:
Hierunter werden Ergebnisse aus der aktuellen medizinischen Forschung verstanden.

c) Register:
Hier werden Krankheitsdaten langfristig gesammelt. Das Ziel ist es, Behandlungsmöglichkeiten weiterzuentwickeln und die Verteilung der Krankheit zu beobachten. Außerdem steht hier, wie man sich als Patient/in registrieren lassen kann.

3. Aktuelle Veranstaltungen

Hierunter fallen gemeinsame Maßnahmen von Patienten und Gesundheitspersonal, um Informationen auszutauschen oder die Erkrankung ins Bewusstsein der Öffentlichkeit zu rücken (z.B.: Termine von Aktionstagen wie dem Tag der Seltenen Erkrankungen).

4. Soziale Beratungs- und Hilfsangebote

Hiermit sind Kontaktdaten und Informationen zu Beratungsstellen gemeint, die Menschen mit seltenen Erkrankungen weiterhelfen können. Darunter fallen ganz unterschiedliche Bereiche, wie Hilfe zu Anträgen und Rechtsansprüchen, aber auch Informationen und Beratung zu psychischen Belastungen und der Selbsthilfe.

a) **Sozialrechtliche Beratung:**
Hier werden Fragen zu Krankenkassenleistungen, Arbeitsrecht, Rentenversicherung und andere beantwortet. Man kann sich hier über alle möglichen Anträge und Erstattungsfragen informieren.

b) **Psychosoziale Beratung:**
Hier finden Sie Informationen und Kontakte zu Beratungsangeboten bei erkrankungsbedingten Problemen innerhalb der Familie, im Freundeskreis oder am Arbeitsplatz zeigen.

c) **Selbsthilfe:**
- *Persönliche Kontakte:* Hier gibt es Kontaktinformationen zu Selbsthilfegruppen, die sich in Ihrer Umgebung treffen und an denen teilgenommen werden kann.
- *Online Kontakte:* An dieser Stelle finden Sie Verweise zu Homepages, Foren im Internet und E-Mailadressen, über die sich Betroffene austauschen können. Es gibt Erfahrungsberichte von anderen Menschen mit seltenen Erkrankungen oder Angehörigen.

ZIPSE
portal-se.de

Gefördert durch: Bundesministerium für Gesundheit aufgrund eines Beschlusses des Deutschen Bundestages

Fragebogen Teil 1

ID

Beschreibung des Bewertungsmaßstabs

Auf den folgenden Seiten werden Ihnen jeweils zwei Informationsbereiche oder –arten gegenübergestellt, deren Wichtigkeit Sie anhand des u.a. Maßstabs bewerten können.

Ausprägungen	Interpretation
1	Informationen sind gleich wichtig
3	Information ist etwas wichtiger
5	Information ist erheblich wichtiger
7	Information ist sehr viel wichtiger
9	Information ist absolut wichtiger

Hier zunächst ein rein ***fiktives Beispiel***:

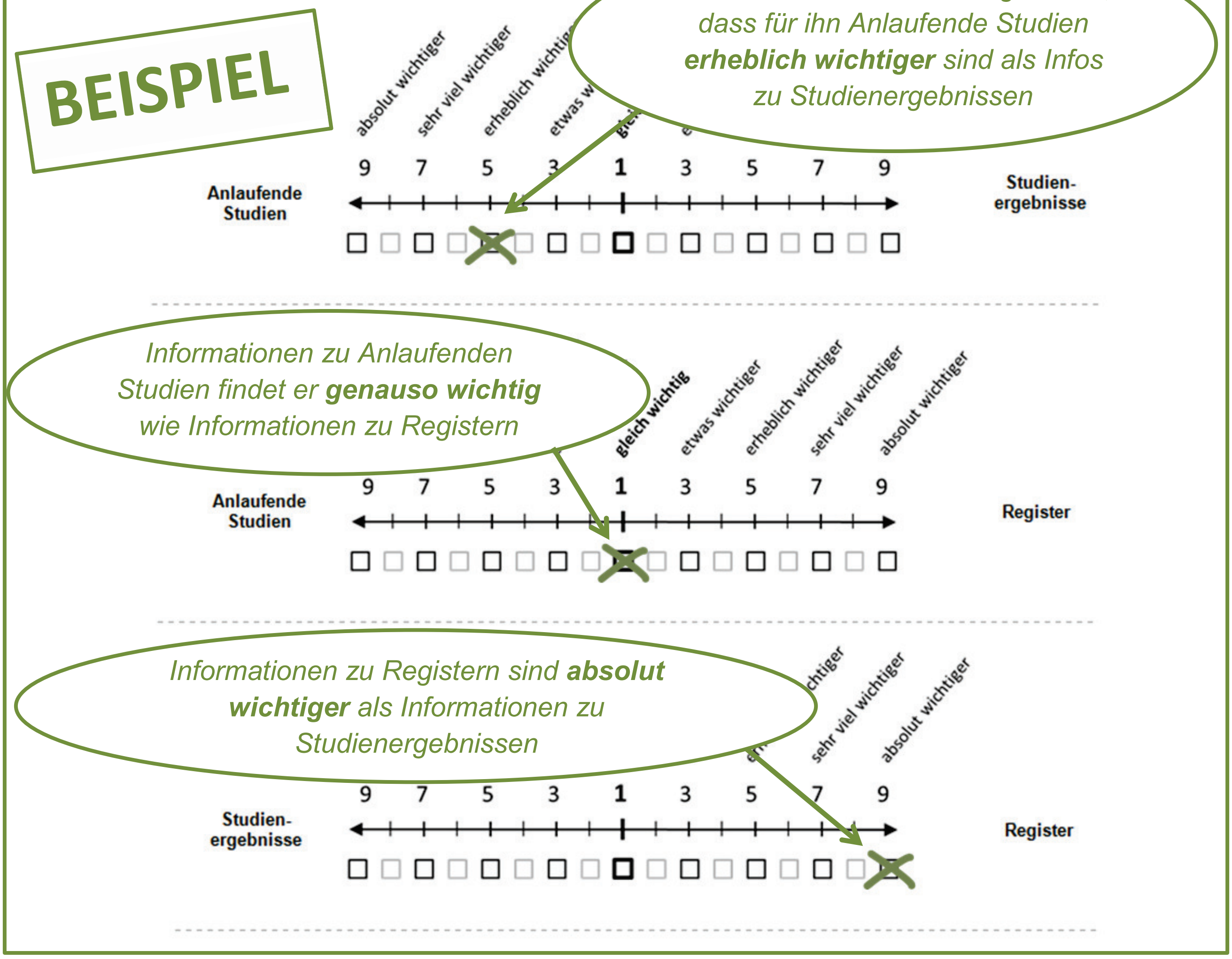

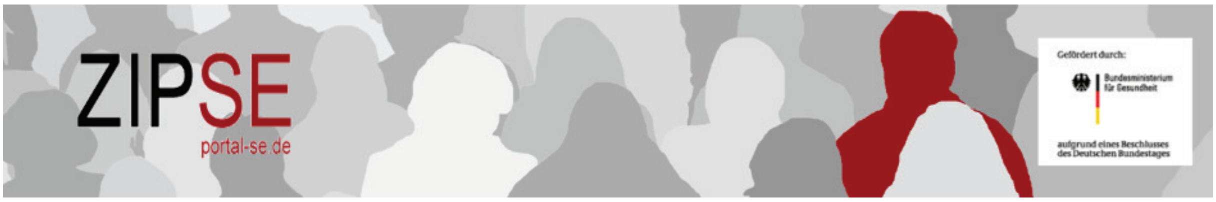

Bitte beginnen Sie nun mit Ihrer Bewertung!

*Bitte nehmen Sie sich nun Zeit folgende Fragen aus Ihrer **ganz persönlichen Sicht** zu beantworten. Bitte fragen Sie sich hierbei, welche Informationen **zum aktuellen Zeitpunkt in Ihrer aktuellen Situation für Sie** wichtiger sind. Es gibt keine falschen Antworten!*
*Überlegen Sie sich zuerst, **welche** der Informationen wichtiger ist und dann **um wie viel** wichtiger die Information ist!*

Wie wichtig sind Ihnen Informationen zu **Medizinischen Fragen**
im Vergleich zu Informationen zur **Forschung**?

absolut wichtiger | sehr viel wichtiger | erheblich wichtiger | etwas wichtiger | **gleich wichtig** | etwas wichtiger | erheblich wichtiger | sehr viel wichtiger | absolut wichtiger

Medizinische Fragen 9 7 5 3 **1** 3 5 7 9 **Forschung**

☐ ☐ ☐ ☐ ☐ ☐ ☐ ☐ ☐ ☐ ☐ ☐ ☐ ☐ ☐ ☐ ☐

Wie wichtig sind Ihnen Informationen zu **Medizinischen Fragen**
im Vergleich zu Informationen zu **Aktuellen Veranstaltungen**?

absolut wichtiger | sehr viel wichtiger | erheblich wichtiger | etwas wichtiger | **gleich wichtig** | etwas wichtiger | erheblich wichtiger | sehr viel wichtiger | absolut wichtiger

Medizinische Fragen 9 7 5 3 **1** 3 5 7 9 **Aktuelle Veranstal-tungen**

☐ ☐ ☐ ☐ ☐ ☐ ☐ ☐ ☐ ☐ ☐ ☐ ☐ ☐ ☐ ☐ ☐

Wie wichtig sind Ihnen Informationen zu **Medizinischen Fragen** im
Vergleich zu **Sozialen Beratungs- und Hilfsangeboten**?

absolut wichtiger | sehr viel wichtiger | erheblich wichtiger | etwas wichtiger | **gleich wichtig** | etwas wichtiger | erheblich wichtiger | sehr viel wichtiger | absolut wichtiger

Medizinische Fragen 9 7 5 3 **1** 3 5 7 9 **Soziale Beratungs- und Hilfs-angebote**

☐ ☐ ☐ ☐ ☐ ☐ ☐ ☐ ☐ ☐ ☐ ☐ ☐ ☐ ☐ ☐ ☐

Wie wichtig sind Ihnen Informationen zur **Forschung** im Vergleich zu Informationen über **Aktuelle Veranstaltungen**?

	absolut wichtiger	sehr viel wichtiger	erheblich wichtiger	etwas wichtiger	**gleich wichtig**	etwas wichtiger	erheblich wichtiger	sehr viel wichtiger	absolut wichtiger	
	9	7	5	3	**1**	3	5	7	9	
**Forschung**	☐	☐	☐	☐	☐	☐	☐	☐	☐	**Aktuelle Veranstaltungen**

Wie wichtig sind Ihnen Informationen zur **Forschung** im Vergleich zu Informationen über **Soziale Beratungs- und Hilfsangebote**?

	absolut wichtiger	sehr viel wichtiger	erheblich wichtiger	etwas wichtiger	**gleich wichtig**	etwas wichtiger	erheblich wichtiger	sehr viel wichtiger	absolut wichtiger	
	9	7	5	3	**1**	3	5	7	9	
**Forschung**	☐	☐	☐	☐	☐	☐	☐	☐	☐	**Soziale Beratungs- und Hilfsangebote**

Wie wichtig sind Ihnen Informationen zu Aktuellen **Veranstaltungen** im Vergleich zu Informationen über **Soziale Beratungs- und Hilfsangebote**?

	absolut wichtiger	sehr viel wichtiger	erheblich wichtiger	etwas wichtiger	**gleich wichtig**	etwas wichtiger	erheblich wichtiger	sehr viel wichtiger	absolut wichtiger	
	9	7	5	3	**1**	3	5	7	9	
**Aktuelle Veranstaltungen**	☐	☐	☐	☐	☐	☐	☐	☐	☐	**Soziale Beratungs- und Hilfsangebote**

Wie wichtig sind Ihnen Informationen zur **Diagnosefindung** im Vergleich zu Informationen zur **Therapie**?

	absolut wichtiger	sehr viel wichtiger	erheblich wichtiger	etwas wichtiger	**gleich wichtig**	etwas wichtiger	erheblich wichtiger	sehr viel wichtiger	absolut wichtiger	
	9	7	5	3	**1**	3	5	7	9	
**Diagnose-findung**	☐ ☐	☐ ☐	☐ ☐	☐ ☐	☐	☐ ☐	☐ ☐	☐ ☐	☐ ☐	**Therapie**

Wie wichtig sind Ihnen Informationen zur **Diagnosefindung** im Vergleich zu **Allgemeinen Informationen zum Krankheitsbild**?

	absolut wichtiger	sehr viel wichtiger	erheblich wichtiger	etwas wichtiger	**gleich wichtig**	etwas wichtiger	erheblich wichtiger	sehr viel wichtiger	absolut wichtiger	
	9	7	5	3	**1**	3	5	7	9	
**Diagnose-findung**	☐ ☐	☐ ☐	☐ ☐	☐ ☐	☐	☐ ☐	☐ ☐	☐ ☐	☐ ☐	**Allge-meines zum Krank-heitsbild**

Wie wichtig sind Ihnen Informationen zur **Therapie** im Vergleich zu Informationen zu **Allgemeinen Informationen zum Krankheitsbild**?

	absolut wichtiger	sehr viel wichtiger	erheblich wichtiger	etwas wichtiger	**gleich wichtig**	etwas wichtiger	erheblich wichtiger	sehr viel wichtiger	absolut wichtiger	
	9	7	5	3	**1**	3	5	7	9	
**Therapie**	☐ ☐	☐ ☐	☐ ☐	☐ ☐	☐	☐ ☐	☐ ☐	☐ ☐	☐ ☐	**Allge-meines zum Krank-heitsbild**

Wie wichtig sind Ihnen Informationen zu **Anlaufenden Studien** im Vergleich zu Informationen zu **Studienergebnissen**?

	absolut wichtiger	sehr viel wichtiger	erheblich wichtiger	etwas wichtiger	**gleich wichtig**	etwas wichtiger	erheblich wichtiger	sehr viel wichtiger	absolut wichtiger	
**Anlaufende Studien**	9	7	5	3	**1**	3	5	7	9	**Studien-ergebnisse**

☐ ☐ ☐ ☐ ☐ ☐ ☐ ☐ ☐ ☐ ☐ ☐ ☐ ☐ ☐ ☐ ☐

Wie wichtig sind Ihnen Informationen zu **Anlaufenden Studien** im Vergleich zu Informationen über **Register**?

	absolut wichtiger	sehr viel wichtiger	erheblich wichtiger	etwas wichtiger	**gleich wichtig**	etwas wichtiger	erheblich wichtiger	sehr viel wichtiger	absolut wichtiger	
**Anlaufende Studien**	9	7	5	3	**1**	3	5	7	9	**Register**

☐ ☐ ☐ ☐ ☐ ☐ ☐ ☐ ☐ ☐ ☐ ☐ ☐ ☐ ☐ ☐ ☐

Wie wichtig sind Ihnen Informationen zu **Studienergebnissen** im Vergleich zu Informationen über **Register**?

	absolut wichtiger	sehr viel wichtiger	erheblich wichtiger	etwas wichtiger	**gleich wichtig**	etwas wichtiger	erheblich wichtiger	sehr viel wichtiger	absolut wichtiger	
**Studien-ergebnisse**	9	7	5	3	**1**	3	5	7	9	**Register**

☐ ☐ ☐ ☐ ☐ ☐ ☐ ☐ ☐ ☐ ☐ ☐ ☐ ☐ ☐ ☐ ☐

Wie wichtig sind Ihnen Informationen zur **Sozialrechtlichen Beratung** im Vergleich zu Informationen über **Psychosoziale Beratung**?

	absolut wichtiger	sehr viel wichtiger	erheblich wichtiger	etwas wichtiger	**gleich wichtig**	etwas wichtiger	erheblich wichtiger	sehr viel wichtiger	absolut wichtiger	
**Sozialrechtliche Beratung**	9	7	5	3	**1**	3	5	7	9	**Psycho-soziale Beratung**

Wie wichtig sind Ihnen Informationen zur **Sozialrechtlichen Beratung** im Vergleich zu Informationen über **Selbsthilfe**?

	absolut wichtiger	sehr viel wichtiger	erheblich wichtiger	etwas wichtiger	**gleich wichtig**	etwas wichtiger	erheblich wichtiger	sehr viel wichtiger	absolut wichtiger	
**Sozialrechtliche Beratung**	9	7	5	3	**1**	3	5	7	9	**Selbsthilfe**

Wie wichtig sind Ihnen Informationen zur **Psychosozialen Beratung** im Vergleich zu Informationen über **Selbsthilfe**?

	absolut wichtiger	sehr viel wichtiger	erheblich wichtiger	etwas wichtiger	**gleich wichtig**	etwas wichtiger	erheblich wichtiger	sehr viel wichtiger	absolut wichtiger	
**Psychosoziale Beratung**	9	7	5	3	**1**	3	5	7	9	**Selbsthilfe**
